# Characterization of Individual Human Antibodies That Bind Pertussis Toxin Stimulated by Acellular Immunization

**DOI:** 10.1128/IAI.00004-18

**Published:** 2018-05-22

**Authors:** Edith Acquaye-Seedah, Elizabeth E. Reczek, Hugh H. Russell, Andrea M. DiVenere, Sara O. Sandman, Joseph H. Collins, Caitlin A. Stein, Timothy A. Whitehead, Jennifer A. Maynard

**Affiliations:** a Department of Biochemistry, The University of Texas at Austin, Austin, Texas, USA; b Excelimmune, Inc., Woburn, Massachusetts, USA; c McKetta Department of Chemical Engineering, The University of Texas at Austin, Austin, Texas, USA; d Department of Chemical Engineering and Materials Science, Michigan State University, East Lansing, Michigan, USA; e Department of Biosystems and Agricultural Engineering, Michigan State University, East Lansing, Michigan, USA; f Institute for Quantitative Health Science and Engineering, Michigan State University, East Lansing, Michigan, USA; Georgia Institute of Technology School of Biological Sciences and Emory University School of Medicine Cystic Fibrosis Center

**Keywords:** Bordetella pertussis, pertussis toxin, plasmablast, B cells, antibodies, epitope, neutralizing antibody, Bordetella, whooping cough

## Abstract

Despite high vaccination rates, the incidence of whooping cough has steadily been increasing in developing countries for several decades. The current acellular pertussis (aP) vaccines all include the major protective antigen pertussis toxin (PTx) and are safer, but they appear to be less protective than infection or older, whole-cell vaccines. To better understand the attributes of individual antibodies stimulated by aP, we isolated plasmablast clones recognizing PTx after booster immunization of two donors. Five unique antibody sequences recognizing native PTx were recovered and expressed as recombinant human IgG1 antibodies. The antibodies all bind different epitopes on the PTx S1 subunit, B oligomer, or S1-B subunit interface, and just one clone neutralized PTx in an *in vitro* assay. To better understand the epitopes bound by the nonneutralizing S1-subunit antibodies, comprehensive mutagenesis with yeast display provided a detailed map of the epitope recognized by antibodies A8 and E12. Residue R76 is required for antibody A8 binding and is present on the S1 surface but is only partially exposed in the holotoxin, providing a structural explanation for A8's inability to neutralize holotoxin. The B-subunit-specific antibody D8 inhibited PTx binding to a model receptor and neutralized PTx *in vitro* as well as in an *in vivo* leukocytosis assay. This is the first study, to our knowledge, to identify individual human antibodies stimulated by the acellular pertussis vaccine and demonstrates the feasibility of using these approaches to address outstanding issues in pertussis vaccinology, including mechanisms of accelerated waning of protective immunity despite repeated aP immunization.

## INTRODUCTION

The current rise in Bordetella pertussis cases has been attributed to various causes, including increased awareness and antigenic divergence between vaccine and circulating clinical strains (1). However, the pertussis resurgence also coincided with the introduction of acellular pertussis (aP) vaccines, raising concerns that the aP-induced immunity may be suboptimal or may wane more quickly than protection induced by natural infection or the previously used inactivated whole-cell vaccine (2). Vaccination with aP prevents the severe manifestations of disease but appears to be unable to eliminate subclinical infection or transmission (3).

Of the numerous toxins produced by Bordetella, the pertussis toxin (PTx) is a 105-kDa extracellular toxin and colonizing factor that is produced only by B. pertussis. The closely related species B. bronchiseptica and B. parapertussis rarely cause disease in humans and contain a transcriptionally silent PTx operon due to promoter mutations (4). PTx belongs to the AB_5_ class of toxins, which are characterized by a catalytically active A subunit (also called S1; 26 kDa) and a receptor-binding B oligomer. This heteropentamer consists of four noncovalently linked subunits, namely, S2 (22 kDa), S3 (22 kDa), S4 (12 kDa), and S5 (11 kDa), at a molar ratio of 1:1:2:1 (5). The B oligomer is responsible for binding host cell surface receptors and mediating internalization of the S1 subunit.

Upon binding to cell surface receptors, PTx is endocytosed into early endosomes, followed by retrograde transport to the Golgi apparatus and then the endoplasmic reticulum (ER). There, upon reduction of a disulfide bond in S1 and an ATP-induced conformational change in the B subunit, S1 is released from the B oligomer (6). After translocation of the unfolded S1 subunit into the cytoplasm, this subunit catalyzes ADP-ribosylation of membrane-associated G_i/o_ proteins, disrupting G-protein signaling (7) and compromising neutrophil/macrophage activities (8). Independent of the toxic S1 activities, the B oligomer also has nonenzymatic effects, including T cell activation and mitogenicity (9). The PTx holotoxin is directly responsible for leukocytosis, which is predictive of clinical outcomes.

While there is no serological correlate of protection for pertussis, studies have shown that high anti-PTx antibody titers after household exposure or immunization correlate with a reduced incidence of severe pertussis (10). However, antibody titers to PTx after aP vaccination have a higher rate of decay than those for other diseases (11), and antibodies recognizing protective epitopes appear to be preferentially elicited by natural infection (12). To better understand the biochemical attributes of individual antibodies responding to PTx after aP vaccination, we applied modern immunological tools to isolate antibody sequences from single B cells stimulated by adult aP booster vaccination. We report the sequences and detailed biochemical analyses (relative binding affinity, *in vitro* neutralization, and epitope specificity) of anti-PTx antibodies recovered from two adults. This work supports the feasibility of applying these approaches to issues in pertussis vaccinology in future studies.

## RESULTS

### Sequence analysis of isolated PTx-specific antibodies.

**FIG 1:**
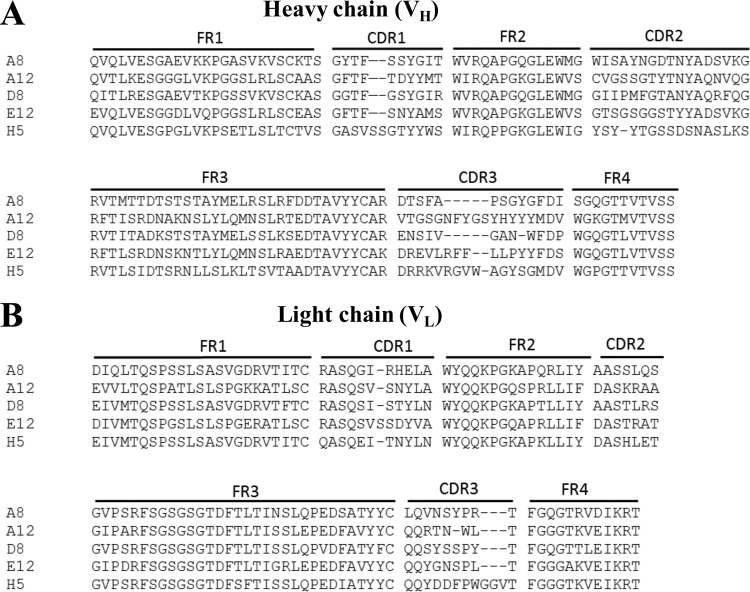
Sequence diversity of the five isolated anti-PTx antibodies. We created an alignment of the amino acid sequences of the anti-PTx antibody variable regions isolated from adults boosted with acellular pertussis vaccines. Heavy (A) and light (B) variable chain sequences were aligned using the online ClustalOmega software (http://www.ebi.ac.uk/Tools/msa/clustalo/).

To profile the human anti-PTx response after aP vaccination, antibody-secreting plasmablasts were isolated from two healthy adult donors 7 days after boosting with an acellular pertussis (aP) vaccine (Adacel or Boostrix). Current acellular vaccines include PTx which has been detoxified chemically by formaldehyde or glutaraldehyde treatment (13), but we used native PTx to identify clones more likely to be relevant during infection. Plasmablast cells were isolated by flow cytometry and sorted into single wells of 96-well plates (50 plates per donor), and the antibody-containing supernatants were screened by a custom Luminex assay for PTx reactivity (see Table S1 in the supplemental material). The antibody variable heavy chain (V_H_) and variable light chain (V_L_) sequences were then amplified from PTx-reactive wells by use of established methodologies (14, 15), resulting in identification of five unique antibody sequences (Fig. 1). Information connecting the isolated sequences to the administered vaccine is not available. Enzyme-linked immunosorbent spot (ELISpot) analyses have suggested that pertussis-specific B cells occur at a rate of 10 to 20 antigen-specific cells per 10^5^ antibody-secreting cells, consistent with this low yield (16).

We first evaluated the germ line sequence usage and level of somatic hypermutation in the variable regions of the isolated antibodies. Candidate parental germ line sequences of the five isolated antibodies were identified by an NCBI/IgBLAST search, and germ line sequences with the highest scores were selected (Table 1). The number of mutations observed in the variable heavy or light (V_H_ or V_K_) gene segments compared to the closest germ line genes ranged from 11 to 24 (Table 1). This is similar to the range observed after influenza vaccination, where 77% of plasmablast B cell clones contained 10 to 29 somatic mutations compared to the germ line genes (17). The length of heavy chain complementarity-determining region loop three (CDR3), a key contributor to antigen binding specificity, ranged from 12 to 18 amino acids, with an average of 15.2 residues.

**TABLE 1:** Candidate parental germ line genes of isolated anti-PTx antibodies

Antibody	Chain	V gene	D gene	J gene	No. of mutations (V, D, J)
A12	Light	IGKV3-11*01		IGKJ4*01	11, 1
E12	Light	IGKV3-20*01		IGKJ4*01	22, 2
A8	Light	IGKV1-17*01		IGKJ1*01	12, 2
H5	Light	IGKV1-33*01, IGKV1D-33*01		IGKJ4*01	11, 0
D8	Light	IGKV1-39*01, IGKV1D-39*01		IGKJ2*01	11, 2
A12	Heavy	IGHV3-11*06	IGHD3-9*01	IGHJ6*03	11, 0, 2
E12	Heavy	IGHV3-23*04	IGHD3-3*01	IGHJ4*02	12, 0, 1
A8	Heavy	IGHV1-18*01	IGHD3-3*02	IGHJ6*02	19, 0, 1
H5	Heavy	IGHV4-61*01	IGHD3-10*01	IGHJ6*02	24, 0, 2
D8	Heavy	IGHV1-69*06, IGHV1-69*14	IGHD1-26*01	IGHJ5*02	13, 0, 0

### Anti-PTx antibodies bind different PTx subunits.

**FIG 2:**
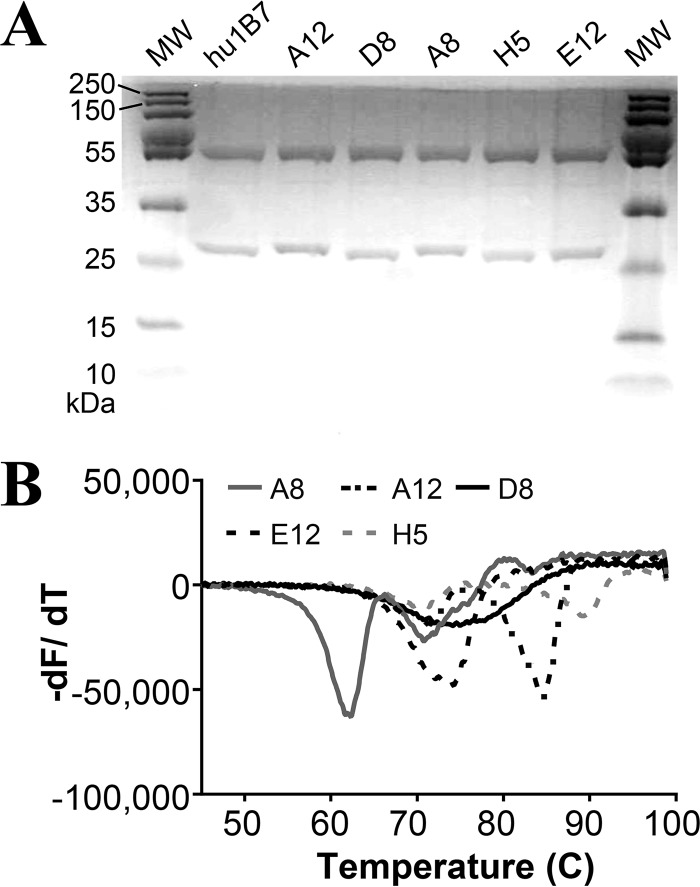
Production of recombinant anti-PTx antibodies. (A) Purity and size of purified IgG antibodies. Protein (3 μg) samples were run under reducing SDS-PAGE conditions, and the sizes of molecular size (MW) markers are indicated. (B) Melting temperature profiles of the purified recombinant antibodies, determined by differential scanning fluorimetry. The curves shown are the averages for four replicates. The melting temperature (*T_m_*) for each antibody was determined from the minimum of the transition temperatures in the derivative data (−dF/dT) and is shown in Table 1.

The antibodies were expressed as recombinant human IgG1 proteins in CHO cells, with the native heavy and light chain pairing, followed by purification by protein A chromatography for further characterization (Fig. 2A). Thermal stability was assessed using differential scanning fluorimetry (Fig. 2B; Table 2). For antibodies with bimodal unfolding, the first transition typically represents unfolding of the C_H_2 domain, while the second transition represents unfolding of the C_H_3 and Fab domains (18). Antibodies A12 and H5 exhibited typical melting curves, with the first transition at ∼70°C and second transitions near 84°C and 89°C, respectively. Antibody A8 appeared to be less stable, with the first transition near 62°C and the second near 72°C. Antibodies D8 and E12 appeared to unfold with a single transition, with a melting temperature (*T_m_*) of ∼74°C, which is suggestive of cooperative unfolding (18).

**TABLE 2:** Biochemical characterization of isolated antibodies and controls^*a*^

Antibody	EC_50_ (nM)				*T_m_* (°C)
	PTx	PTg	PTx S1-220	PTx B oligomer	
A12	0.10 ± 0.03	0.18 ± 0.02	ND	ND	70.69 ± 0.21 (*T_m_*1), 84.59 ± 0.24 (*T_m_*2)
E12	0.13 ± 0.006	0.05 ± 0.02	0.05 ± 0.03	ND	74.27 ± 0.08 (*T_m_*1)
A8	0.15 ± 0.003	0.3 ± 0.08	0.02 ± 0.006	ND	62.06 ± 0.68 (*T_m_*1), 72.29 ± 2.52 (*T_m_*2)
D8	0.14 ± 0.04	0.11 ± 0.08	ND	0.78 ± 0.22	74.05 ± 1.00 (*T_m_*1)
H5	0.02 ± 0.01	0.06 ± 0.03	ND	0.11 ± 0.05	70.69 ± 0.74 (*T_m_*1), 89.34 ± 0.70 (*T_m_*2)
hu1B7	0.06 ± 0.01	0.07 ± 0.03^*b*^	0.008 ± 0.002	ND	ND
hu11E6	0.17 ± 0.03	0.06 ± 0.007^*b*^	ND	1.18 ± 0.19	ND
P-IVIG	4.26 ± 1.27	1.05 ± 0.04	9.39 ± 1.67	ND	ND

a Data are means ± SE. ND, not determined.

b A murine antibody was used.

**FIG 3:**
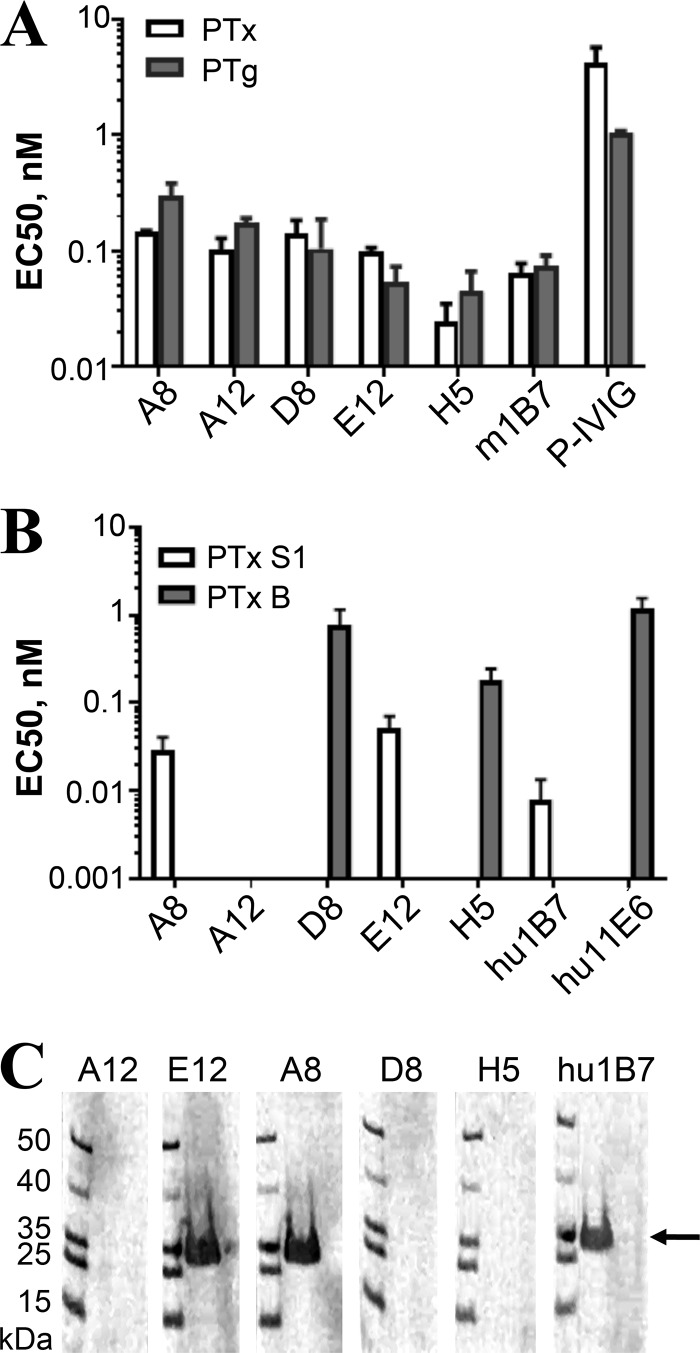
Binding activities of anti-PTx antibodies. (A) ELISA was used to assess the binding of the monoclonal antibodies to native PTx and genetically modified PTx (PTg). ELISAs were performed at least twice, with similar results. Error bars represent standard errors for replicate experiments. Due to availability, the murine 1B7 antibody was assessed for binding to PTg, while the rest of the antibodies have a human constant domain. (B) Binding specificities of the isolated anti-PTx antibodies for purified S1 and B subunits, as assessed by ELISA. The gap for A12 indicates no detectable binding to either subunit, suggesting that it binds an epitope spanning the subunit interface. (C) Binding of the antibodies to purified PTx S1 on a Western immunoblot. Assays were performed in parallel, with equal loading of prestained molecular size markers and PTx (500 ng) onto SDS-PAGE gels. After transfer, the strips were separated, probed with the indicated primary antibodies, and then combined for staining with the secondary antibodies, detection, and imaging. The experiment was repeated at least three times with identical results.

We then assessed the ability of the five human antibodies to recognize native and genetically modified PTx (PTg). PTg contains two amino acid substitutions (R9K and E129G) in the enzymatically active S1 subunit and has been proposed as a replacement for chemically detoxified PTx (PTd) in current acellular vaccines due to its reduced toxicity in conjunction with increased stability and immunogenicity (19). A previous study observed various differences in binding of some antibodies to PTx or PTg, suggesting that the R9K and E129G amino acid changes in PTg may induce small conformational changes which may affect the epitopes of certain antibodies (20). We compared the 50% effective concentrations (EC_50_s) for antibody binding to PTx and PTg by using an enzyme-linked immunosorbent assay (ELISA). The well-characterized S1-specific antibody hu1B7, which consists of humanized variable domains with human IgG1/κ constant domains (21), was used as a control. The antibodies bound native PTx and PTg at low nanomolar concentrations, with similar EC_50_s (Fig. 3A; Table 2). Binding levels with the control antibody m1B7 (21, 22) were also similar for PTx and PTg.

We next assessed the EC_50_ for binding to either the PTx S1 subunit or B oligomer by using an ELISA (Fig. 3B; Table 1). For the S1 subunit antigen, we used PTx S1-220, a soluble, recombinant version of the S1 subunit with similar properties (23). The control antibody hu1B7, whose affinity for PTx was previously determined to be 1.2 nM by competition ELISA and BIAcore analysis (21), bound PTx S1-220 at subnanomolar EC_50_s. Antibodies A8 and E12 strongly bound PTx S1-220 with similar subnanomolar EC_50_s. In contrast, antibody A12 appeared to weakly bind PTx S1-220 at antibody concentrations near 0.6 μM, while no binding was observed for antibodies D8 and H5 at concentrations of up to 67 nM. The control antibody hu11E6, which binds the S2 and S3 subunits with a previously determined affinity of 7 nM (21), bound the purified PTx B oligomer with a low nanomolar EC_50_. Only antibodies D8 and H5 showed strong binding, while A8, A12, and E12 did not bind to the PTx B oligomer at antibody concentrations of up to 67 nM. The inability of A12 to bind either PTx S1-220 or the B oligomer, despite strong binding to intact PTx in the ELISA, suggests that its epitope spans the S1 and B subunits.

The subunit specificity of the antibodies was further assessed by Western blotting. PTx dissociates into its component five subunits under reducing and denaturing SDS-PAGE conditions, resulting in a loss of conformational epitopes. Antibodies A8 and E12 revealed a band of ∼26 kDa on the Western blots, suggesting that there are linear elements to their binding epitopes (Fig. 3C). Antibody hu1B7 is known to bind S1 on Western blots (23) and recognized a band of similar size, supporting the idea that A8 and E12 bind the S1 subunit. Binding of A12, D8, and H5 to PTx subunits was not detected, suggesting that they bind purely conformational epitopes.

### Anti-PTx antibodies bind unique epitopes.

**FIG 4:**
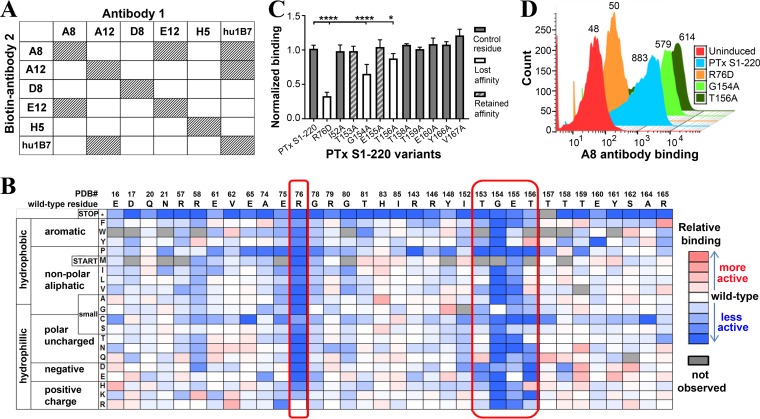
Characterization of the A8 epitope on PTx S1-220. (A) Epitope binning of isolated antibodies by competition ELISA. PTx was used to coat ELISA wells, followed by the addition of a biotinylated human antibody and an unlabeled competitor antibody at various concentrations. Bound biotinylated antibody was detected by use of streptavidin-HRP. Results for each antibody pair are from at least one ELISA performed with duplicate antibody samples. (B) Subset of the fitness metric heat map for A8 IgG binding to yeast-displayed PTx S1-220 variants. Deep sequencing and entropy analysis of the variants selected from A8 IgG binding to a PTx S1-220 mutagenesis library implicated candidate residues (circled sections) predicted to be involved in binding. (C) Validation of A8 candidate epitope residues identified from deep sequencing of yeast-displayed PTx S1-220 variants. Binding of A8 IgG to the yeast-displayed PTx S1-220 variants was assessed by ELISA. Data are represented as normalized binding relative to the wild-type level (*A*_450, variant_/*A*_450, wild type_) at an antibody concentration of 2 μg/ml, so lower values indicate reduced binding. The means were compared to that for wild-type PTx S1-220 by using ANOVA and Tukey's test (α = 0.05; *, *P* ≤ 0.05; ****, *P* < 0.0001). (D) Representative flow cytometry histogram of A8 antibody binding to yeast display variants. The mean fluorescence intensities are shown next to the peaks.

To further map the epitopes, we performed competitive binding experiments with control antibodies hu1B7 and murine 11E6 (m11E6). hu1B7 binds an epitope on the S1 subunit of PTx (22–24) and was able to inhibit binding of antibodies hu1B7 and A12 when it was added as a biotinylated competitor, suggesting that A12 binds an epitope similar to that bound by hu1B7 (Fig. 4A; Fig. S2A). Antibody 11E6 binds the S2 and S3 subunits of the PTx B oligomer, is thought to interfere with PTx binding to receptors, and protects against PTx activities *in vitro* and *in vivo* (22). Murine 11E6 did not prevent binding of any of the five antibodies to PTx, suggesting that the antibodies bind epitopes that are distinct from the 11E6 epitope (Fig. S2B).

The five isolated antibodies were then tested for competition with each other by use of biotinylated and unlabeled antibodies (Fig. 4A; Fig. S2C to G). In this format, each of the biotinylated antibodies inhibited binding of its unlabeled form to PTx, as expected. Biotinylated A12 also competed with hu1B7 for binding to PTx, providing additional evidence of overlap between the A12 and hu1B7 epitopes. Biotinylated A8 inhibited unlabeled E12 binding to PTx, and biotinylated E12 also inhibited A8 binding to PTx, suggesting that the A8 and E12 epitopes are adjacent to each other. Biotinylated A8 also inhibited hu1B7 binding to PTx, although we did not observe inhibition of A8 by biotinylated hu1B7. This may reflect the higher affinity of hu1B7 (Fig. 3A; Table 2) and/or suggests that the A8 epitope may partially overlap the hu1B7 epitope or that A8 binding may sterically inhibit hu1B7 binding. This was further tested in subsequent experiments. Antibodies D8 and H5 did not inhibit binding of any of the other antibodies to PTx, suggesting that they bind unique epitopes.

### Antibodies A8 and E12 bind epitopes close to the neutralizing 1B7 epitope.

Antibody hu1B7 potently neutralizes PTx *in vivo*, preventing PTx-driven leukocytosis (21). Thus, information about the epitopes of anti-PTx antibodies can help to define structural requirements for PTx neutralization. The A12 antibody seems to span the A subunit and the B oligomer, making its epitope more challenging to elucidate. Similarly, the detailed epitopes of the B-oligomer antibodies D8 and H5 were more difficult to determine, as the four subunits comprising the B oligomer have not been expressed as a folded unit in Escherichia coli and introducing these changes into the B. pertussis chromosome presents other challenges. Based on these limitations, we further characterized the epitopes of the S1 subunit binding antibodies (A8 and E12).

The hu1B7 epitope was previously mapped at the single-residue level by use of yeast-displayed libraries of the truncated PTx S1-220 construct (23, 24). We therefore used the single-site saturation mutagenesis library of PTx S1-220 to map the A8 and E12 epitopes (25). The yeast library was induced for surface expression of PTx S1-220 and labeled with biotinylated IgG at half the experimentally determined yeast affinity, followed by streptavidin-phycoerythrin. Surface display of PTx S1-220 was monitored via a C-terminal c-myc epitope (by use of anti-c-myc–fluorescein isothiocyanate [FITC]). Cells in a gated population (400,000) were collected from a single fluorescence-activated cell sorter (FACS) sort (Fig. S3A; Table S2). Deep sequencing was then used to determine enrichment ratios for the bound and displayed populations compared to the initial library population.

Sorting with an IgG antibody was found to be less sensitive than previously observed for the hu1B7 Fab fragment (24). Sequence entropies were therefore compared for hu1B7 Fab or IgG binding to PTx S1-220; epitope residues were accurately identified by using a new cutoff of <75% of the sequence entropy range as well as requiring the residue to have a solvent-accessible surface area of >20% for the IgG sorts (rather than <50% for the Fab sorts) (Fig. S3B). Using this new cutoff, the following five conserved residues for A8 binding to PTx S1-220 were identified: R76, T153, G154, E155, and T156 (Fig. 4B). These residues are spatially contiguous on the crystal structure of PTx, which is consistent with their forming the core of a conformational epitope.

To validate the experimentally determined epitope residues, we analyzed binding of the antibodies to yeast-displayed variants of PTx S1-220 containing a single residue change at a putative epitope position. Alanine variants of the identified residues were tested, with the exception of the R76D mutation, which was tested as a charge reversal mutation to minimize the effects of a large difference in size. Additional alanine variants were tested for control residues close to but not expected to form the A8 epitope, as follows: I152A, T158A, T159A, E160A, Y166A, and V167A. Binding of the IgG to yeast-displayed PTx S1-220 variants was then assessed by titrating the antibody in ELISA wells coated with induced yeast at an optical density at 600 nm (OD_600_) of 0.5, with relative binding for each variant assessed as the ratio of wild-type PTx S1-220 to variant binding. Binding of A8 to the R76D, G154A, and T156A variants was approximately 30, 65, and 88%, respectively, compared to that to wild-type PTx S1-220 (Fig. 4C). This implicated R76 and G154 as key residues involved in the A8 epitope. To further validate the identified residues, antibody binding to the PTx S1-220 variants was assessed by flow cytometry. There was a marked loss of A8 binding to the R76D variant, reflected by the median fluorescence intensity (MFI) of antibody binding with the R76D variant being similar to that of the uninduced control (Fig. 4D).

To exclude the possibility that differences in display levels of the PTx S1-220 variants influenced these results, we monitored display by use of an antibody binding the C-terminal c-myc tag and flow cytometry. Compared to the control unstained yeast cells, display of the R76D variant was slightly reduced, while the G154A variant appeared to be displayed better (Fig. S4A). Due to the slightly reduced display of the R76D variant, we compared binding of 3F10, a previously described weakly neutralizing anti-PTx antibody (22), to that of the variants as an additional control. We observed that A8-biotin did not compete with 3F10 for PTx binding (Fig. S4B), suggesting that these antibodies bind distinct epitopes and thus that 3F10 presents a good control. Antibody 3F10 exhibited low-affinity binding to the R76D and T156A variants that was similar to that of wild-type PTx S1-220 (Fig. S4C). These results suggest that reduced binding of A8 to the R76D and G154A variants is largely due to involvement of these residues in forming the epitope, not to reduced surface display or an altered conformation of the variants.

To evaluate the E12 epitope, a similar approach was utilized. The following seven conserved residues which were spatially contiguous on the crystal structure of PTx were identified for E12: T153, G154, E155, T156, T158, T159, and E160 (Fig. S5). Alanine variants of the identified residues were generated, as well as control residues close to but not expected to form the E12 epitope: R76D, I152A, Y166A, and V167A. Analysis with E12 binding to yeast-displayed PTx variants suggested that the G154 and E160 residues help to form the E12 epitope (Fig. S4). The smaller differences of E12 IgG binding to the candidate residues limits the strength of these conclusions.

**FIG 5:**
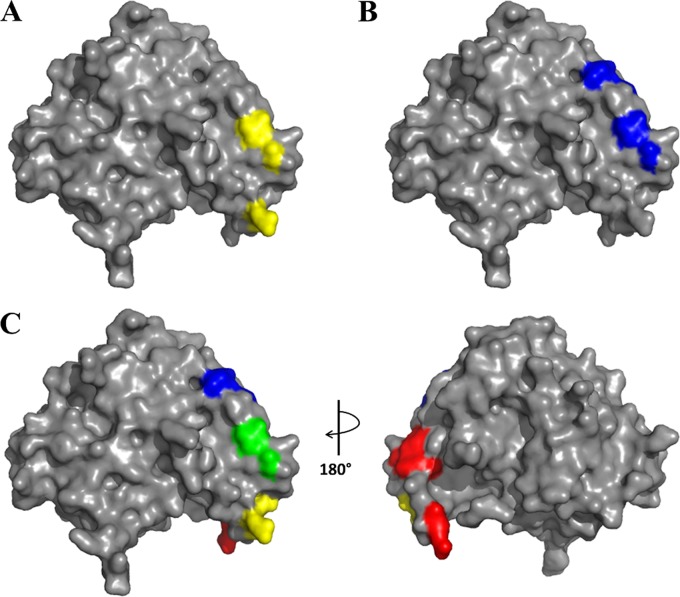
Comparison of A8 and E12 epitopes on PTx. Experimentally determined conformational epitopes for A8 (yellow) (A) and E12 (blue) (B) are shown. (C) Rotated views (90°) of the PTx S1 crystal structure (PDB entry 1PRT), with conserved residues indicated for A8 (yellow), E12 (blue), overlapping A8 and E12 (green), and hu1B7 (red) (23, 24).

From the results described above, the A8 epitope is at the base of the S1 subunit, consisting of one of three antiparallel β-sheets and an α-helix contiguous to the S4 subunit, and is distal to the catalytic PTx S1 residues (His35 and Glu129) (26). The combination of the competition assays and mutational analysis suggested that E12 binds close to the A8 epitope, and the locations of the validated epitope residues are close to the identified hu1B7 epitope (Fig. 5; Fig. S6). While the 1B7 epitope (23, 24) appears to be readily accessible for antibody binding, the key residue for A8 binding, R76, is sandwiched between the S4 and S5 subunits of the B oligomer (Fig. S6B), suggesting that A8 may bind the dissociated S1 subunit more readily. These results further support use of the yeast-displayed PTx S1-220 library (24) as a platform for epitope mapping of other neutralizing and nonneutralizing anti-PTx S1-subunit antibodies.

### D8 neutralizes PTx toxicity *in vitro*.

Observations that aP vaccination confers a limited duration of immunity against disease (27) and elicits higher titers of antibodies recognizing PTd rather than native PTx (12) led to the hypothesis that aP vaccination results in poorer antibody quality and corresponding poorer long-term protection due to “original antigenic sin.” As such, we tested the five anti-PTx antibodies for neutralizing activity against PTx by using *in vitro* and *in vivo* assays.

**FIG 6:**
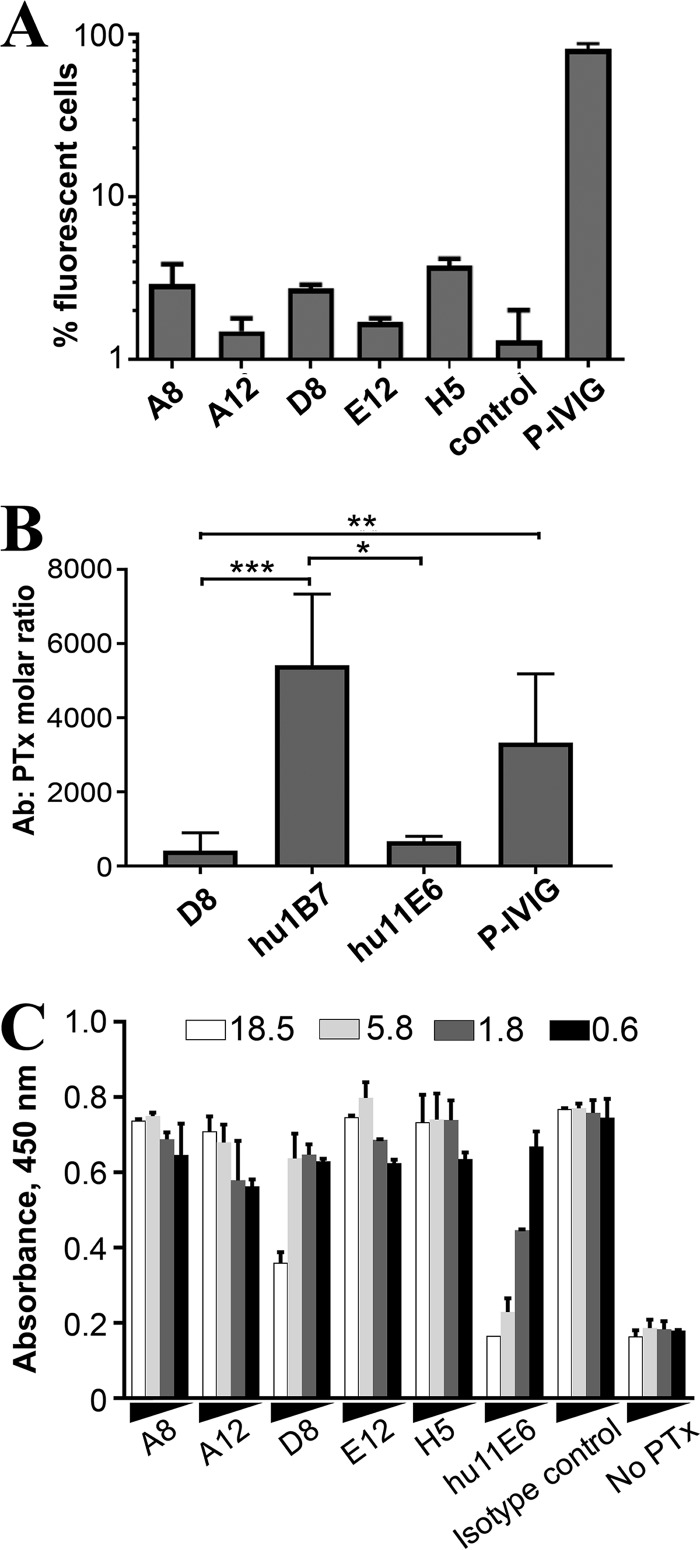
Antibody neutralization of PTx *in vitro*. (A) Anti-PTx antibody binding to whole-cell B. pertussis monitored by flow cytometry. Antibodies were incubated with mid-log-phase bacteria and detected with anti-human IgG. Data are presented as means, and error bars represent standard deviations of the means. (B) *In vitro* inhibition of PTx-mediated clustering of CHO-K1 cells. Results are presented as the means for 6 replicates, and error bars represent the standard deviations from the means. Statistical significance was determined using Kruskal-Wallace analysis and Dunn's multiple-comparison test (α = 0.05; *, *P* < 0.05; **, *P* < 0.01, ***, *P* < 0.001). (C) Antibody-mediated blockade of PTx-receptor binding, with fetuin as a model receptor. Wells were coated with fetuin and blocked. Antibody and PTx were preincubated at the indicated molar ratios (molar excess of antibody ranging from 0.6× to 18.5×) and added to the blocked wells. PTx was detected with a murine anti-PTx antibody cocktail, followed by anti-mouse IgG–HRP and TMB substrate. The hu11E6 antibody, known to block PTx-receptor interactions, was used as a positive control, while wells lacking PTx were used as a negative control. The ELISA was performed twice, with similar results; error bars indicated are the standard errors of the means.

Although PTx is a secreted toxin, PTx molecules are transiently associated with the bacterial surface during secretion (28). Thus, any surface-exposed PTx on whole bacteria may potentially serve as a target for antibody-mediated bactericidal activity (29). Binding of the antibodies to whole bacteria was therefore assessed by incubating the antibodies with live B. pertussis, washing off unbound antibody, and detecting bound antibody with a fluorescent secondary antibody by flow cytometry analysis. We detected no significant binding of the anti-PTx antibodies to B. pertussis (Fig. 6A), suggesting that the antibodies are unlikely to mediate bactericidal activity. Accordingly, none of the antibodies showed any bactericidal activity in a complement killing assay (data not shown).

Next, we analyzed the ability of the antibodies to directly neutralize PTx toxicity *in vitro*. In the presence of PTx, eukaryotic CHO-K1 cells grow in a clustered morphology (30), which can be rescued to a normal elongated, nonclustered morphology in the presence of neutralizing anti-PTx antibodies (22, 31). Of the five antibodies, only D8 inhibited PTx-mediated CHO cell toxicity. In this assay, D8 was more potent than the potently neutralizing anti-PTx antibody hu1B7 (*P* = 0.0003) or the human polyclonal anti-PTx preparation P-IVIG (human intravenous pertussis immunoglobulin) (*P* = 0.0041) (Fig. 6B).

A possible mechanism of toxin neutralization for the B-subunit-specific D8 antibody is competitive inhibition of toxin-receptor binding. To evaluate this, we first determined the ability of the antibodies to inhibit PTx-receptor interactions in a solid-phase binding assay with the serum glycoprotein fetuin as a model receptor. PTx promiscuously binds proteins with terminally sialylated oligosaccharide sequences *in vitro*. These are found on glycoproteins, such as transferrin, haptoglobin, and fetuin, making these useful model receptors for *in vitro* studies, including cocrystallization (32). PTx was first incubated with serially diluted antibody and then added to ELISA plates coated with fetuin. Immobilized PTx was then detected using a mixture of murine anti-PTx antibodies. Of the five antibodies, only D8 inhibited PTx binding to fetuin (Fig. 6C), albeit less effectively than the control antibody, hu11E6 (22). An isotype control antibody showed the maximal PTx-fetuin binding response, while controls lacking PTx reflected the minimal response.

### D8 neutralizes PTx toxicity *in vivo*.

Next, the ability of antibody D8 to protect against PTx-induced leukocytosis *in vivo* was evaluated (31). Antibody D8 was selected for its ability to neutralize toxicity in the CHO assay (Fig. 6B) and to reduce PTx binding to the model receptor fetuin (Fig. 6C). Antibodies that do not neutralize toxicity in the *in vitro* CHO cell assay do not neutralize it in the mouse model, but the CHO cell assay is less reliable at predicting *in vivo* neutralization (33). We therefore assessed the ability of D8 to protect against PTx effects by using an *in vivo* toxin neutralization assay. This assay was performed because it is technically simpler than infection, and protection in this assay was shown to correlate closely with protection against bacterial infection for a set of >30 different anti-PTx antibodies (34).

**FIG 7:**
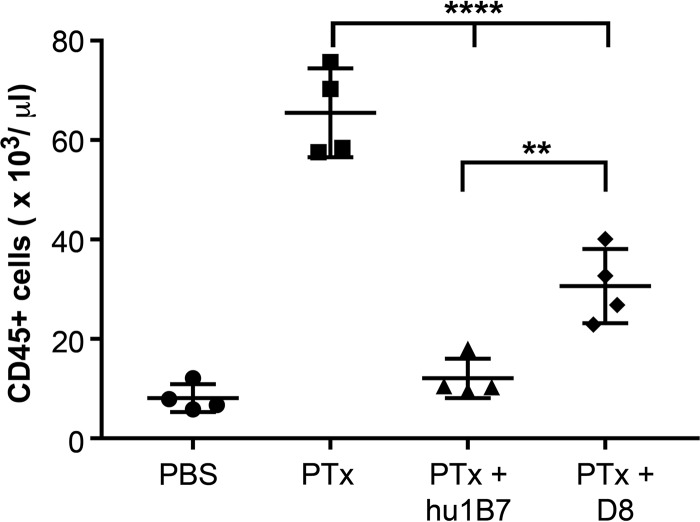
Antibody D8 suppresses PTx-induced leukocytosis in mice. Mice (groups of 4) were administered PBS, 2 μg PTx, or 2 μg PTx plus 20 μg antibody by subcutaneous injection in a 100-μl volume. Four days later, blood was harvested by cardiac puncture, and CD45^+^ leukocytes were measured by flow cytometry. Groups were compared using ANOVA and Tukey's test (α = 0.05; **, *P* < 0.01; ****, *P* < 0.0001).

Mice (*n* = 4) were administered phosphate-buffered saline (PBS) or 2 μg of PTx, either alone or preequilibrated with 20 μg antibody. Four days later, whole blood was collected by cardiac puncture, with the CD45^+^ white blood cell count measured by flow cytometry. Control mice administered PBS only retained a low CD45^+^ white blood cell count (8,127 ± 2,804), while mice administered PTx only showed a significant increase in leukocytosis (65,484 ± 8,944) (*P* < 0.0001), as previously observed (31). The positive-control antibody hu1B7 significantly reduced the white blood cell count compared to that for PTx-only-treated mice (12,074 ± 3,943) (*P* < 0.0001) but did not significantly change the white blood cell count compared to that for PBS-treated mice, consistent with previous reports (31). Antibody D8 also significantly reduced leukocytosis compared to that of PTx-treated mice (30,628 ± 7,493) (*P* < 0.0001), although less effectively than the result with hu1B7 (*P* = 0.006) (Fig. 7). Treatment with antibody A8, which was nonneutralizing in the CHO assay, did not significantly reduce leukocytosis at the 20-μg antibody dose tested (data not shown).

## DISCUSSION

Recent developments in immunoprofiling have provided unprecedented insight into antigen-specific antibody repertoires, facilitating identification of antibodies binding rare, neutralizing epitopes and design of immunogens to elicit neutralizing antibody responses. These technologies rely on isolation of donor antigen-specific B cells, from which the antibody variable region genes are sequenced, individually cloned, and expressed recombinantly for biochemical and *in vitro* characterization. The sequences of antibodies isolated from plasmablasts, precursors to the mature bone marrow plasma cells responsible for the bulk of the serum antibodies, correlate with those of serum antibodies observed by use of proteomics, indicating that these approaches identify the specific sequences present in the serum responsible for ELISA signals observed during serological analyses (35). These approaches have resulted in identification of antibodies neutralizing diseases, such as HIV and influenza, and have helped us to understand the impacts of vaccination and natural exposure in development of protective antibody responses (36, 37). In the present study, we applied these technologies to pertussis.

Epidemiological data suggest that the current acellular pertussis vaccines confer a more limited duration of protection than that with older, whole-cell vaccines (38) and are a major contributing factor to the current rise in observed clinical pertussis cases (39). Interestingly, while the duration of protection appears to be shorter, aP vaccination induces higher initial serum titers against pertussis antigens, which drop more slowly than those induced by whole-cell pertussis (wP) vaccines or natural infection (40). Compared to those for other toxin-based vaccines, anti-pertussis serum titers decay more rapidly: 6 to 12 months for pertussis versus 19 years for tetanus (41). The immunologic mechanisms responsible for enhanced protection conferred by whole-cell vaccines have not been elucidated fully but are crucial to the development of appropriate countermeasures and next-generation vaccines with enhanced efficacy (42).

While there is no accepted clinical correlate of disease, several lines of evidence support PTx as a key protective antigen as well as the ability of anti-PTx antibodies to confer protection. Elevated levels of neutralizing anti-PTx antibodies correlate with protection against severe disease in humans (43). PTx-only acellular vaccines are used in Denmark and Thailand and have been proposed for use during maternal vaccination. Along these lines, a recent study demonstrated that a high-dose PTx-only monocomponent vaccine (20 μg PTx with 0.5 mg Alhydrogel) given to pregnant baboons protected the resulting neonates. These infants were experimentally infected at 5 weeks of age, when they exhibited low but measurable anti-PTx titers. Animals with vaccinated mothers exhibited significantly reduced symptoms (leukocytosis and clinical observations) compared to those of animals with unvaccinated mothers, despite similarly high levels of bacterial colonization (44). Similarly, adolescent baboons administered a cocktail of two anti-PTx antibodies 3 days after experimental infection exhibited a rapid improvement in leukocytosis compared to that for untreated control animals (21). Collectively, these data raise the questions of how to induce long-lasting protective antibody responses against PTx and what role antibody and epitope diversity plays in protection.

As an initial study to better understand the B cell responses elicited by aP immunization, we identified unique PTx-reactive antibody sequences from plasmablasts of two recently boosted adult donors. We focused on responses to PTx, as this toxin mediates many of the symptoms associated with severe pertussis infection (45) and induces an antibody response which contributes significantly to protection (46). Expression of the sequences as recombinant antibodies enabled us to characterize the PTx epitopes recognized by these clones and their likely roles in protection. Of the five antibodies identified, two recognize the S1 subunit, two recognize the B subunit, and one appears to bind an epitope spanning the S1-B subunit interface. One antibody recognizes a novel epitope on the B subunit and neutralizes PTx activities *in vitro* and *in vivo*.

The epitopes recognized by the previously characterized neutralizing antibodies, hu1B7 and hu11E6, are protective in mice and baboons (21). Of the antibodies reported in this work, A8 and A12 competed with hu1B7 for binding to PTx, suggesting that their epitopes are adjacent to or overlap the hu1B7 epitope. The reduction in A8 binding to fetuin-bound PTx (see Fig. S7 in the supplemental material) suggests an inability to bind receptor-bound holotoxin. However, unlike hu1B7, antibodies A8 and A12 did not neutralize PTx-mediated CHO cell toxicity, and antibody A8 was nonneutralizing in a murine leukocytosis assay. The mechanisms of PTx neutralization by anti-S1-subunit antibodies are not well understood (23); thus, epitope mapping of other neutralizing and nonneutralizing PTx S1 antibodies will prove informative in terms of better understanding the underlying structural determinants for the effective neutralization of PTx toxicity.

Both the catalytically active PTx S1 subunit (34) and the B oligomer can elicit potently neutralizing antibodies (47). Based on competition ELISAs, the anti-B-subunit antibodies D8, H5, and hu11E6 all bind separate epitopes. Antibody D8 potently neutralized PTx-mediated CHO cell clustering, with a potency similar to that of hu11E6 and more potent than that of hu1B7, but was less effective than hu11E6 in preventing PTx-receptor interactions. This antibody was also able to neutralize PTx activities *in vivo*, though to a lesser extent than that for hu1B7. Notably, hu11E6 is also more protective in the CHO cell assay but is less protective than hu1B7 in mice (21, 31). Due to the challenges in expressing B-oligomer subunits, we have not identified the D8 epitope with greater precision, although an epitope that occludes the receptor binding site on PTx is consistent with our data.

These results have broad implications for the development of improved acellular pertussis vaccines. First, consistent with ELISpot data, we observed a small number of unique anti-PTx antibody sequences. While the desirable level of sequence diversity is unknown, a combination of just two anti-PTx antibodies can exhibit synergy in protection, supporting the idea that at least some diversity in epitope recognition is beneficial (31). Anti-PTx titers may be elevated by future vaccine formulations which boost the total number of reactive B cells as well as the number of B cell lineages with distinct sequences. For instance, human serum titers can be elevated by high-dose PTx formulations and inclusion of the genetically (as opposed to chemically) detoxified pertussis toxin (48); thus, it would be important to know whether PTg vaccination also increases anti-PTx sequence diversity. Second, just one of these five antibodies neutralized PTx. This is a key feature in disease protection, since nonneutralizing antibodies are not protective against infection. Current acellular vaccines contain chemically detoxified PTx; the detoxification procedure stabilizes and inactivates PTx but also slightly modifies the protein structure and surface epitopes. Analyses of serum antibody responses indicate that PTd induces antibodies which preferentially recognize PTd over native PTx (20). As PTg retains a more native-like structure, it will be important to determine whether it induces a higher fraction of antibody sequences that recognize native PTx and neutralize toxin activities. Single-cell profiling strategies, such as those used here, provide novel tools for understanding these differences and the effects of vaccine formulation changes on the resulting antibodies in greater detail. Third, we defined the epitopes of several new vaccine-relevant anti-pertussis antibodies. This helps us to understand which antibodies are likely to be beneficial and can guide formulation and engineering of PTx to preserve these epitopes, as has been achieved for other protective antigens (49).

The anti-PTx antibodies isolated in this study were likely influenced by several factors. Both donors were adults who received the wP vaccine as infants, which includes little free PTx, but either this or prior exposure to B. pertussis may have biased their subsequent responses to aP booster vaccines. Typically, upon secondary antigenic exposure, such as after booster vaccination or reinfection, reactivation and rapid proliferation of preexisting specific memory B cells can lead to large numbers of short-lived antibody-secreting plasmablasts which appear in the circulation (50). In a previous study, ∼100 unique tetanus toxin-specific plasmablast clones were identified from two adult donors boosted with tetanus toxoid (51). In the current study, just five unique PTx-specific antibodies were found, despite screening of 50 96-well plates of plasmablasts. It is unclear to what extent this small number of clones reflects the immunosuppressive qualities of PTx, the intrinsic shortcomings of the aP vaccine, or the individual characteristics of the two donors. ELISpot analyses have shown that PTx induces fewer antigen-specific B cells than those induced by other antigens, consistent with our results (16, 52). Future experiments with larger numbers of donors and isolated anti-PTx antibodies will be needed to reach clear conclusions about the nature of PTx-reactive antibodies elicited by currently available aP vaccines.

This work provides a connection between acellular pertussis immunization and the resulting antibody responses to PTx at the sequence level. Most prior clinical trials evaluating pertussis vaccines used serum titers as the primary means to measure an antibody response to a given antigen, with fewer using ELISpot analyses to monitor B cell populations and antigen specificity. While they are simple and readily standardized, serum titers cannot distinguish between a high concentration of low-affinity antibodies and a low concentration of high-affinity antibodies. Similarly, ELISpot assays are unable to assess the clonal diversity of the antibody sequences or to relate a specific B cell to a desirable functional response, such as epitope specificity or protection. In future work, immunoprofiling is likely to be instrumental in determining whether protection against PTx requires antibodies binding a small number of discrete epitopes (e.g., receptor-blocking antibodies, as in the case of tetanus toxin [35]) versus the advantages of a more diverse antibody response and to assess the impacts of formulation changes, such as replacing PTd with PTg or including new adjuvants.

## MATERIALS AND METHODS

### Study design.

Peripheral blood mononuclear cells (PBMCs) from two adult donors were isolated 7 days after immunization. As part of their routine medical care, the donors were boosted with one of two similar acellular pertussis vaccines. Adacel (Sanofi Pasteur) contains 5 flocculation units (Lf) tetanus toxoid, 2.5 Lf diphtheria toxin, 2.5 μg inactivated PTx, 5 μg Fha, 3 μg pertactin, and 5 μg type 2 and 3 fimbriae as antigens and 1.5 mg aluminum phosphate (0.33 mg aluminum) as adjuvant, with ≤5 μg residual formaldehyde, <50 ng residual glutaraldehyde, and 3.3 mg (0.6% [vol/vol]) 2-phenoxyethanol in a 0.5-ml volume. Boostrix (GlaxoSmithKline) contains 5 Lf tetanus toxoid, 2 Lf diphtheria toxin, 8 μg inactivated PTx, 8 μg Fha, and 2.5 μg pertactin, with aluminum hydroxide as adjuvant (not more than 0.39 mg aluminum by assay), 4.5 mg of sodium chloride, ≤100 μg of residual formaldehyde, and ≤100 μg of polysorbate 80 (Tween 80) in a 0.5-ml volume. An institutional review board (IRB)-approved consent form was obtained from each donor, giving permission to collect their blood for research purposes. Human samples were obtained by infectious disease specialists by use of protocol EX002, which was approved by the St. Patrick Hospital/Community Medical Center Joint Institutional Review Board (Missoula, MT).

### Isolation of plasma cells and antibody cloning.

Plasma B cells were enriched from the isolated PBMCs and antibody sequences amplified similarly to previously established methods (14). Briefly, enrichment of plasma B cells from PBMCs was achieved using a plasma cell isolation kit (Miltenyi Biotec), and cells were labeled with antibodies (BioLegend) to CD19, CD38, and kappa or lambda light chains and single cell sorted by FACS analysis (BD FACSJazz). Single cells were distributed into 96-well PCR plates containing reverse transcriptase (RT) buffer, an RNase inhibitor (NEB), and primers for the heavy chain region and both light chains (kappa and lambda). Reverse transcription was performed via Superscript III RT (Invitrogen). The cDNA was divided, and separate seminested PCRs were performed to produce heavy chain VDJ and light chain VJ amplicons, maintaining cognate pairing of antibodies. For antibody expression, a human IgG1 constant region was grafted onto the cloned heavy and light chain sequences in a single expression plasmid under the control of EF1alpha promoters. To screen the antibodies for antigen binding, the antibodies were transiently expressed in HEK293 mammalian cells (ATCC) in a 96-well format. From the microplates, supernatants were removed and screened for antibody-antigen interactions in custom Luminex assays (see the supplemental material).

### Sequence analyses.

To assess the clonal relatedness of the isolated antibodies, variable genes (heavy and light chains) were amplified from the plasmablasts by RT-PCR. DNA sequences of the variable-gene segment of the isolated antibodies were aligned using ClustalOmega (http://www.ebi.ac.uk/Tools/msa/clustalo/). NCBI/IgBLAST was used to map the VDJ or VJ sequences of each VH or Vκ, respectively, to the closest human germ line sequence from the IMGT database. The level of somatic hypermutation was assessed by selecting the germ line sequence with the highest score, and the number of somatic mutations was noted. CDR-H3 sequences were identified according to the Kabat scheme as the residues between Cys-XXX-XXX (usually Cys-Ala-Arg) and Trp-Gly-XXX-Gly.

### Antibody production.

Antibodies were transiently expressed on a laboratory scale in adherent CHO-K1 cells and purified from the culture supernatant by protein A affinity chromatography as previously described (31). Antibody size and purity were evaluated by 12% SDS-PAGE on gels stained with GelCode Blue (Pierce). Human intravenous pertussis immunoglobulin (P-IGIV) (53) was obtained from the Massachusetts Public Health Biologic Laboratory.

### PTx.

Native PTx (in glycerol or lyophilized), the PTx B oligomer, and genetically modified PTx (PTg) were purchased from List Biological Laboratories, Inc. (Campbell, CA). Native PTx was also obtained through BEI Resources, NIAID, NIH (B. pertussis toxin, salt-free; NR-31826). A soluble, truncated version of the PTx S1 subunit, PTx S1-220, was expressed and purified from the pAK400 plasmid in E. coli, as described previously (23).

### Thermal stability analysis.

The purified recombinant antibodies at a concentration of 150 μg/ml were assayed for thermal stability by use of differential scanning fluorimetry as previously described (31). Antibody fluorescence was quantified using a Viia7 RT-PCR system (Applied Biosystems) at a rate of 0.05°C/s. The melting temperature (*T_m_*) was estimated from the minimum of the plots of the negative first derivative of the fluorescence with respect to temperature (−dF/dT), as described previously (54). Average *T_m_* values were determined for antibody samples run in quadruplicate.

### Antibody binding to PTx and subunits.

The antibody 50% effective binding concentration (EC_50_) for PTx holotoxin or subunits was determined by an indirect PTx ELISA as previously described (21). ELISA plates were coated overnight at 4°C with PTx holotoxin (1 μg/ml), PTx S1-220 (0.25 μg/ml), or PTx B oligomer (0.85 μg/ml). To estimate the EC_50_, the binding curves were fit to the 4-parameter logistic (4PL) nonlinear curve model, using GraphPad Prism 5.

The ability of the antibodies to compete with a biotinylated competitor antibody was evaluated by competition ELISA. 1B7 was biotinylated with a 20× molar excess of sulfo-NHS-biotin (Thermo Scientific) following the manufacturer's instructions. Due to their low concentrations, the recombinant expressed human antibodies were biotinylated with a 1,000× or 5,000× (D8) molar excess of sulfo-NHS-LC-biotin following the manufacturer's instructions. Excess biotin was removed by dialysis against PBS. Antibodies were assessed for incorporation of biotin by use of a capture ELISA for binding to the holotoxin. Biotinylated antibody binding to PTx was detected with streptavidin-horseradish peroxidase (streptavidin-HRP) (BD Pharmingen) at a 1:500 dilution. To assess competition for binding to close or overlapping epitopes, PTx was first used to coat plates overnight at 1 μg/ml. A constant concentration of 0.6 μg/ml of the biotinylated competitor antibody was added to serially diluted antibody starting from 20 μg/ml. The antibody mixtures were then added to the coating of PTx. The biotinylated antibody was detected with a 1:500 dilution of streptavidin-HRP (BD Pharmingen), and the reaction was developed using tetramethylbenzidine (TMB) substrate.

### PTx binding to model receptor fetuin.

Fetuin was used to coat the plates at a concentration of 10 μg/ml in PBS overnight at 4°C and then blocked with PBSTM (PBS-Tween with 5% nonfat dry milk). Serially diluted antibodies in PBSTM were preincubated with a constant concentration of 0.79 nM PTx and added to the fetuin coating. Bound PTx was detected with an equimolar mixture of murine antibodies to PTx (3F10, G9A, 10C9, 1B7, 11E6, and 7F2; all sourced from the National Institute for Biological Standards and Control, United Kingdom), each at 0.1 μg/ml. The mouse antibodies were detected using an HRP-conjugated goat anti-mouse secondary antibody, and the plates were developed with TMB. The absorbance was read at 450 nm, and data were analyzed using GraphPad Prism 5.

### Western blotting.

To detect antibody epitopes on PTx subunits in a Western blot, 0.5 μg PTx was boiled in reducing SDS buffer for 2 min and then run in an SDS (12% [wt/vol])-PAGE gel, and PTx subunits were transferred to a polyvinylidene difluoride (PVDF) membrane. The membrane was blocked with PBSTM, cut into strips containing separated PTx subunits, and incubated with 2.5 μg of antibody. The membranes were then probed with 1:1,000 HRP-conjugated anti-human Fc and Cκ antibodies and developed using a colorimetric TMB substrate kit (Fisher Scientific).

### Flow cytometry analysis of antibody binding to whole B. pertussis.

Bacteria were grown on Bordet-Gengou agar with 15% defibrinated sheep blood (HemoStat Labs), and single clones that showed a characteristic zone of hemolysis around the colonies, indicative of the Bvg^+^ state of the bacteria, were selected for liquid culture. The cells were resuspended in PBS, and the OD_600_ per milliliter was adjusted to 1. The cells were then washed with PBS and incubated with 10 μg of each antibody on ice. After washing with PBS, bound antibodies were detected with a 1:500 dilution of goat anti-human Fc conjugated to Alexa Fluor 647. Negative controls included irrelevant human antibodies as well as secondary antibodies alone incubated with the cells. Events (10,000 per treatment) were collected on a BD SLRII Fortessa flow cytometer. The cell-associated fluorescence for a gated population of the cells was determined using FlowJo_V10 software.

### Yeast display library and sorts.

Anti-PTx S1 IgGs were chemically biotinylated at a molar ratio of 20 mmol biotin to 1 mmol IgG by use of an EZ Link NHS-biotin kit following the manufacturer's instructions (Life Technologies). Observed equilibrium dissociation constants (*K_D_*) of the biotinylated IgGs to yeast-displayed PTx S1-220 were determined using clonal population yeast display titrations according to the method of Chao et al. (55). IgG concentrations of 8 pM to 600 nM were tested.

Conformational epitope mapping closely followed the protocol described by Kowalsky et al. (24, 56). Yeast display sorts were conducted using the previously described PTx S1-220 site saturation mutagenesis library (24). For sorting, cells were stained at an IgG labeling concentration of half the observed *K_D_* and antibody binding detected using fluorescently labeled secondary antibodies. Cells were also probed with an anti c-myc antibody (Miltenyi Biotec) to assess surface display of PTx S1-220. Events (400,000) were sorted for each collected population (see Fig. S3A and Table S2 in the supplemental material). Yeast plasmid DNA was prepared for deep sequencing following the protocol of Kowalsky et al. (56). Library DNA was sequenced on an Illumina MiSeq sequencer by use of a 250 × 2 Illumina MiSeq kit (Illumina, San Diego, CA) at the Michigan State University Sequencing Core. Sequencing data were analyzed following the procedure described by Kowalsky et al. (56). Sorting with an IgG was found to be less sensitive than that with a Fab. Sequence entropies were therefore compared for 1B7 Fab and IgG binding to PTx S1-220, and a new cutoff was introduced for the IgG sorts to accurately identify the epitope residues identified by using an IgG (Fig. S3B).

### Antibody binding to yeast-displayed PTx S1-220 variants.

To validate the epitope residues identified by deep sequencing, desired point mutations were introduced into the PTx S1-220 gene in the pETCON vector by round-the-plasmid PCR (57). Plasmid DNAs were sequenced to verify the correct point mutations and transformed into competent yeast cells prepared using a Frozen-EZ Yeast Transformation II kit (Zymo Research). Transformed yeast cells were then grown for 2 to 3 days at 28 to 30°C on YNB-CAA-glucose plates (6.7 g/liter yeast nitrogen base, 5 g/liter Casamino Acids, 2% glucose, 1× penicillin-streptomycin, and 1.5% agar). To assess antibody binding to yeast-displayed PTx S1-220 variants, individual yeast colonies with wild-type or variant PTx S1-220 in the pETCON vector were inoculated into YNB-CAA-glucose medium without agar and grown overnight at 30°C. The yeast cells were pelleted and resuspended in YNB-CAA-galactose (2%) medium to an OD_600_ of ∼0.75 to 1 and grown further for ∼42 h at 20°C for expression and surface display of PTx S1-220. The induced yeast variants were then washed with PBS, used for coating at an OD_600_ of 0.5, blocked with PBSTM, and incubated with antibody dilutions. Bound antibody was detected by use of goat anti-human Fc–HRP secondary antibody (Thermo Fisher). Data are represented as normalized binding relative to the wild-type level, based on the *A*_450_ at an antibody concentration of 2 μg/ml (*A*_450, variant_/*A*_450, wild type_). To assess surface display of the variants, 2 μg/ml of the A8 and E12 antibodies and 5 μg/ml of the anti-c-myc antibody 9E10 were incubated with the induced yeast variants at an OD_600_ of 0.5/ml for 1 h on ice. The cells were then labeled with fluorescent secondary antibodies and analyzed by flow cytometry. A total of 10,000 events were collected. A8 and E12 binding to the variants is represented by histograms, with the median fluorescence intensity (MFI) of the yeast population indicated. Antibody 9E10, specific for the C-terminal c-myc tag, was used to assess the level of PTx S1-220 display and is represented by histograms, with the MFI of the population with positive antibody binding indicated. The groups were compared by analysis of variance (ANOVA) with Tukey's test.

### PTx structures.

Structures of the PTx holotoxin (PDB entry 1PRT) with the A8, E12, and 1B7 epitopes were generated using PyMOL molecular graphics software.

### Inhibition of PTx-mediated CHO cell clustering.

The CHO neutralization assay was used as described previously (31) to determine the antibody-mediated neutralization of PTx-induced CHO cell clustering. Each antibody was serially diluted in duplicate in a 96-well tissue culture plate, and PTx was added to a final concentration of 5 pM. After preincubation of the antibody-PTx solutions at 37°C for 30 min, freshly trypsinized CHO cells were added at 1.5 × 10^4^ cells/well. Clustering due to PTx toxicity was scored after ∼18 h, as follows: 0, no clustering; 1, a few clusters/equivocal clustering; 2, positive clustering; and 3, maximum/complete clustering. The neutralizing dose was expressed as the molar concentration of antibody which relieved PTx-mediated clustering from a score of 1 to 0.

### *In vivo* leukocytosis assay.

We assessed the neutralizing potential of the antibodies by using an *in vivo* leukocytosis assay essentially as described previously (31). Modifications to the protocol were the use of PTx in glycerol, coincubation of PTx and antibody for 1 h at room temperature, and subcutaneous administration. Statistical analysis was performed using one-way ANOVA and Tukey's test.

Animal procedures were performed in accordance with protocols approved by the University of Texas at Austin (approval no. AUP-2014-00414). The animals were housed and procedures performed at the University of Texas at Austin in a facility approved by the Association for Assessment and Accreditation of Laboratory Animal Care International.

### Accession number(s).

Processed sequencing reads for this work have been deposited as .csv files in figshare (https://figshare.com) under accession numbers doi:10.6084/m9.figshare.5978614, doi:10.6084/m9.figshare.5978596, doi:10.6084/m9.figshare.5978563, doi:10.6084/m9.figshare.5978509, doi:10.6084/m9.figshare.5977987, and doi:10.6084/m9.figshare.5977963.

## Supplementary Material

Supplemental material
