# Supplementary material for: Characterization of Individual Human Antibodies That Bind Pertussis Toxin Stimulated by Acellular Immunization
Source: Infect Immun. 2018 May 22;86(6):e00004-18. doi: 10.1128/IAI.00004-18 (PMC5964521; doi:10.1128/IAI.00004-18)
Supplement: Supplemental material [file supp_86_6_e00004-18__index.html]

Supplemental material 

# Characterization of Individual Human Antibodies That Bind Pertussis Toxin Stimulated by Acellular Immunization

## Supplemental material

- Supplemental file 1 -

  Supplemental methods. Fig. S1. Functional binding of biotinylated antibodies to PTx. Fig. S2. Epitope binding of anti-PTx antibodies in competitive ELISA. Fig. S3. Selection of A8 epitope residues. Fig. S4. Validation of epitope residues with yeast-displayed PTx S1-220 variants. Fig. S5. Characterization of the E12 binding epitope. Fig. S6. Comparison of A8 and hu1B7 epitopes on PTx-S1. Fig. S7. Antibodies bind receptor-bound PTx. Table S1. Representative Luminex data: screening antibodies for PTx specificity. Table S2. Yeast display sorting statistics for A8 and E12 IgG binding from the PTx S1-220 library.

  PDF, 1.3M
