# Supplementary material for: Characterization of Individual Human Antibodies That Bind Pertussis Toxin Stimulated by Acellular Immunization
Source: Infect Immun. 2018 May 22;86(6):e00004-18. doi: 10.1128/IAI.00004-18 (PMC5964521; doi:10.1128/IAI.00004-18)
Supplement: Supplemental material [file IAI.00004-18_zii999092414s1.pdf]

Supplemental methods

Antibody-antigen screening. Luminex™ MagPlex® microspheres were coated with antigens using an xMAP Antibody Coupling Kit according to the manufacturer's instructions (Luminex Corporation). In brief, the desired number of microspheres were placed in a microtube, washed and activated for coupling using the activation buffer. Sulfo-NHS was added, followed by EDC. The microspheres were incubated for 20 minutes at room temperature on a rotator in the dark. The microspheres were washed before coating conditions were added and then incubated for 2 hours at room temperature while rotating. The microspheres were then washed and placed in storage buffer (1% PBS, 1% BSA and 0.05% sodium azide). For all washing steps, a DynaMag™ Magnet (Invitrogen) was used. Each Luminex™ MagPlex® microsphere received a single antigen for coupling. For multiplexing, antigens were coupled to Luminex™ MagPlex® microspheres with unique spectral signatures. Antigens were purchased from List Labs and included Pertussis Toxin (whole), Pertussis Toxin protomer A and Pertussis Toxin protomer B.

Coupled beads were then used to perform the antibody-antigen assays. The assay was run according to manufacturer's instructions (Luminex). Briefly, coupled microspheres were placed in wells of 96 well microplates and incubated with supernatants derived from transfected HEK293 cells for 30 minutes. The microplates were then washed with assay buffer (1X PBS pH 7.4 with 1% BSA) and a PE conjugated goat anti-Fc antibody (Jackson ImmunoResearch) was added and incubated for another 30 minutes. After incubation, the microplates were washed and resuspended in assay buffer. Microplates were then read in a Luminex FlexMap 3D. Reactivities were scored based on values of fold over background (Table S1).

Supplemental data

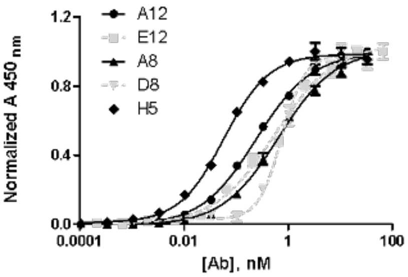

Antibody	Biotinylated mAb EC_{50}	Non-biotinylated mAb EC_{50}
A12	0.24	0.1
E12	0.56	0.13
A8	0.65	0.15
D8	0.76	0.14
H5	0.06	0.02

Figure S1. Functional binding of biotinylated antibodies to PTx. The human antibodies were biotinylated with Sulfo-NHS-LC-Biotin following the manufacturer's instructions. Binding of the serially diluted antibodies to a 1 µg/ml coat of PTx was determined using streptavidin-HRP with TMB detection. The relative binding affinity (EC_{50}) shown next to the curves were determined from fitting the binding curves to the four-parameter logistic (4PL) non-linear curve model using the GraphPad Prism 5 software.

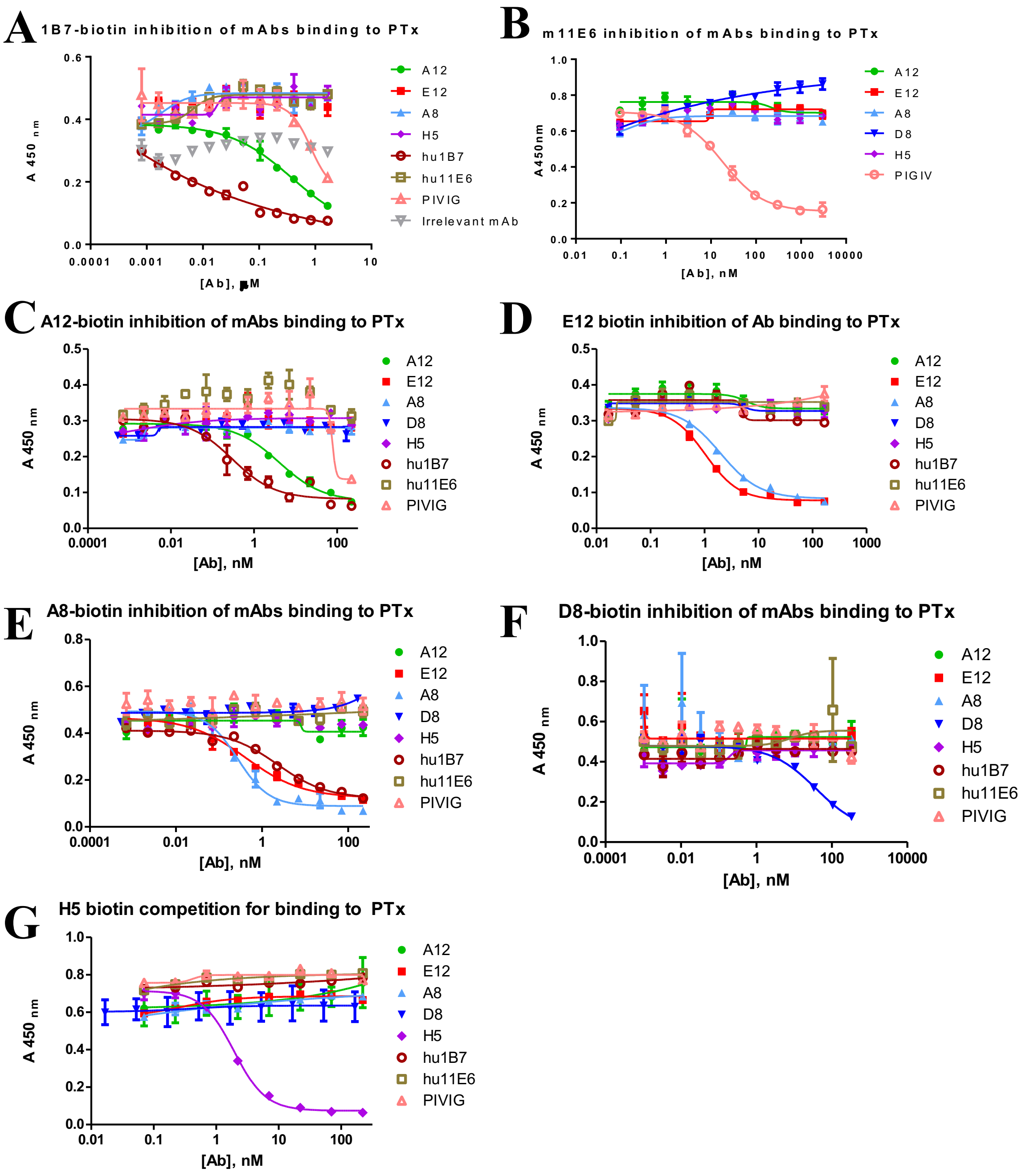

Figure S2. Epitope binning of anti-PTx antibodies in competitive ELISA. ***A, C-G***. Serially diluted human antibodies with 4 nM biotinylated antibody were added to a coat of PTx. Bound biotinylated antibody was detected using streptavidin HRP. ***B,*** Serially diluted human antibodies with a constant concentration of m11E6 antibody were added to a coat of PTx. Bound mouse antibody was detected using an HRP conjugated goat anti-mouse Ig constant kappa chain secondary antibody.

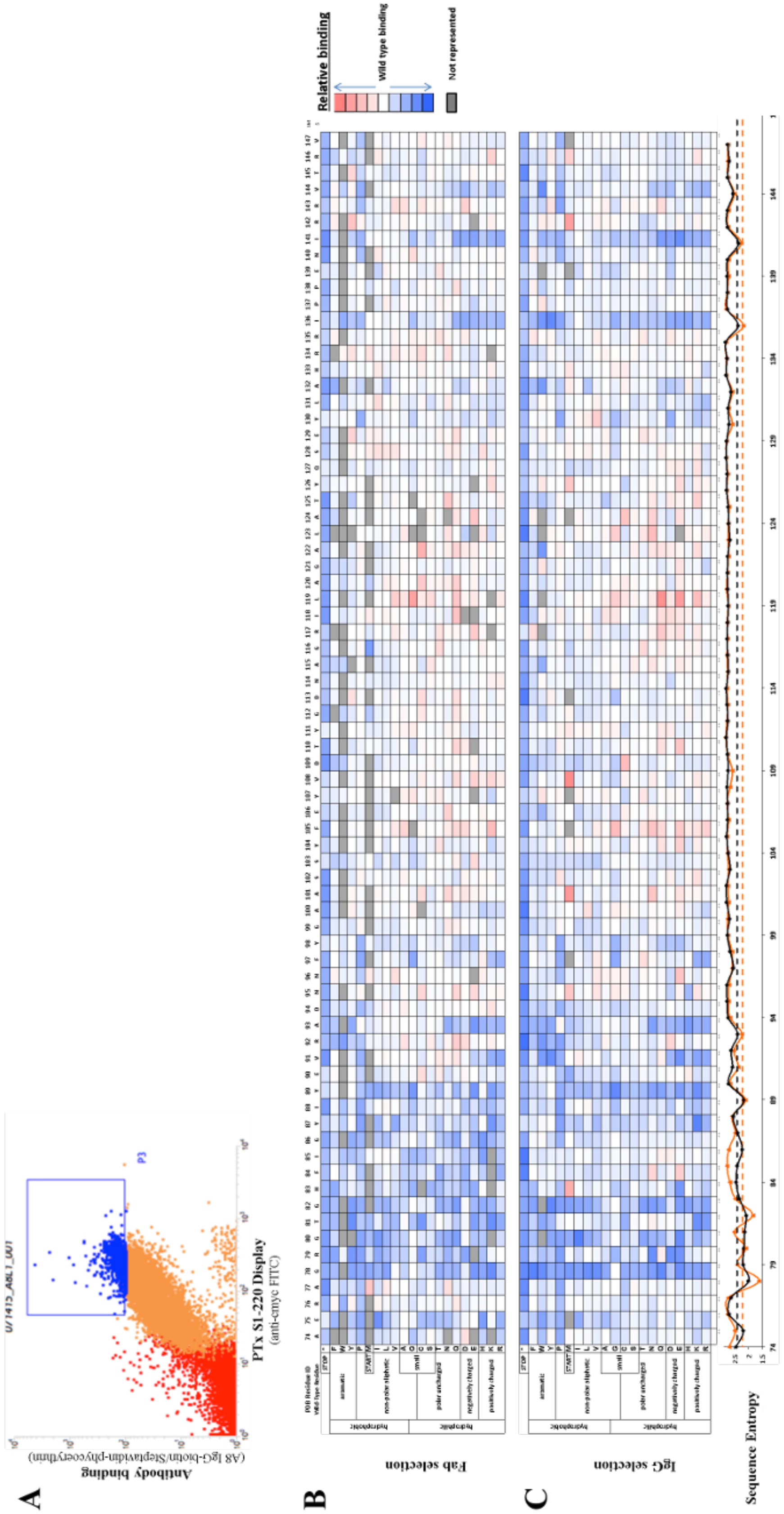
A
Antibody binding
(A8 IgG-biotin/Steptavidin-phycoerythrin)
PTx S1-220 Display
(anti-cmyc FITC)
P3
B
Fab selection
C
IgG selection
Sequence Entropy
Relative binding
Wild type binding
Not represented

Figure S3. Selection of A8 epitope residues. ***A,*** Representative sorting cytogram for yeast displayed PTx S1-220 using biotinylated A8 IgG antibody. The x-axis is the fluorescence channel 1 for display of PTx S1-220, the y-axis is the fluorescence channel 2 for antibody binding. Orange is Fsc/SSc and Fsc/Fl-1 gated population, blue (P3) is the binding gate used in sorts. Sequence-function heatmaps for PTx S1 sorts with hu1B7. Sorting with an IgG is less sensitive then when using a Fab, thus heat maps were compared for epitope mapping experiments for ***B,*** hu1B7 Fab and ***C,*** hu1B7 IgG. The sequence entropy is plotted for Fab (black) and IgG (orange) with their respective cutoffs. A new cutoff was introduced for the IgG sorts to accurately identify the epitope residues identified using a Fab.

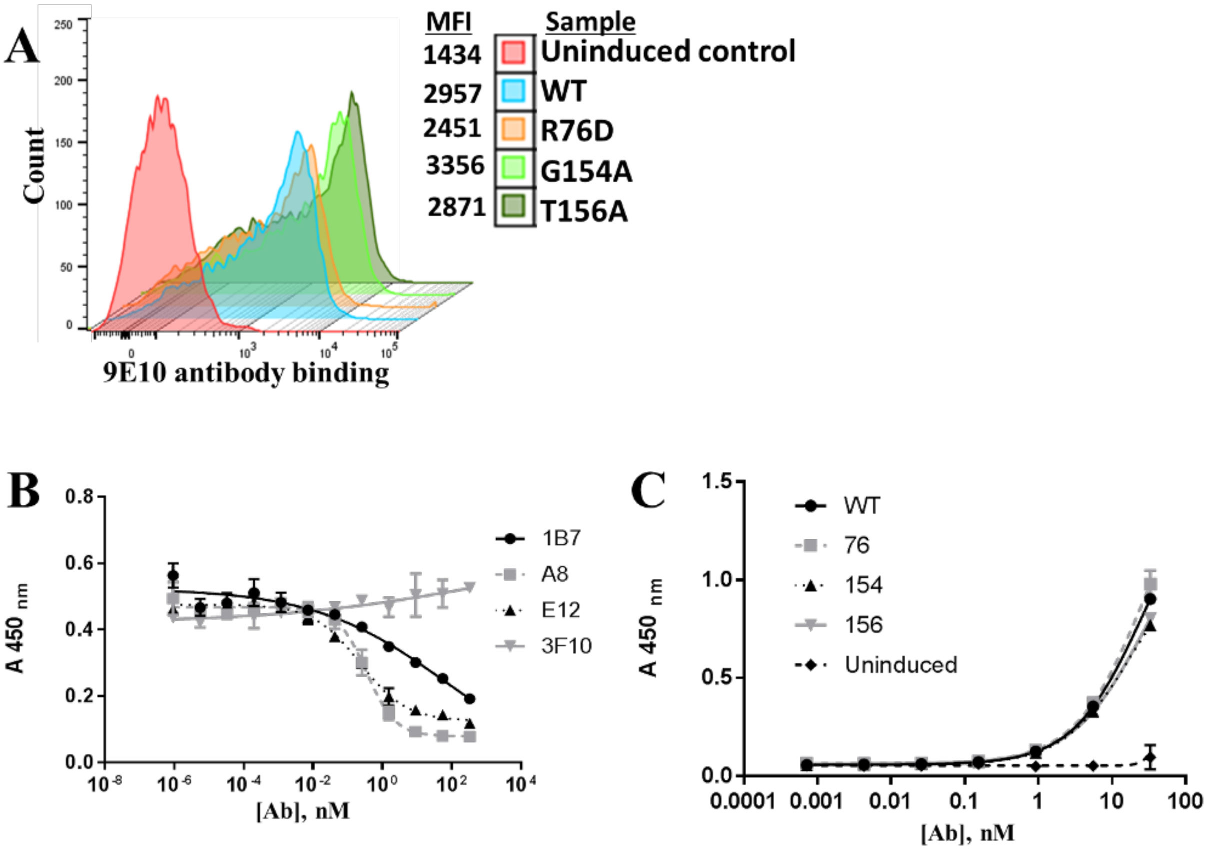

Figure S4. Validation of epitope residues with yeast displayed PTx S1-220 variants. ***A,*** Levels of display on yeast for PTx S1-220 variants. Flow cytometry histograms of yeast displaying different PTx-S1-220 variants detected via the c-myc tag at the PTx c-terminus and anti-c-myc antibody 9E10 followed by fluorescent anti-mouse antibody. The mean fluorescence intensity (MFI) of the population with positive antibody binding is shown. ***B,*** Competitive binding of antibodies to PTx in the presence of 4nM biotinylated A8. PTx was coated on ELISA wells, followed by the addition of biotinylated A8 and unlabeled competitor antibody in varying concentrations. Bound biotinylated antibody was detected with streptavidin-HRP.. ***C,*** Representative ELISA for 3F10 binding to yeast displayed PTx S1-220 variants. Serially diluted 3F10 was added to yeast displayed variants coated at an OD_{600} of 0.5. Antibody binding was detected with anti-mouse Fc HRP.

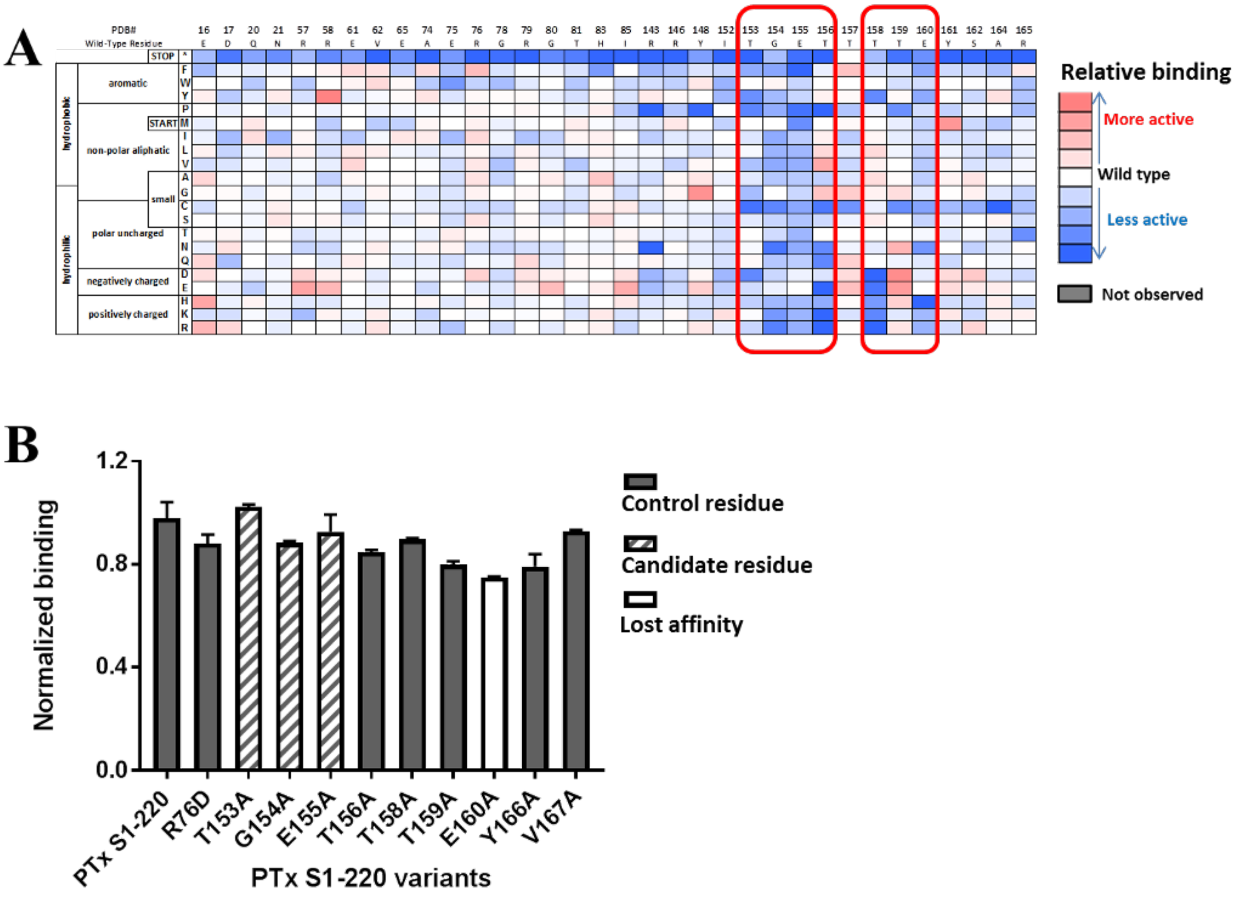

Figure S5. Characterization of the E12 binding epitope. ***A,*** Subset of the fitness-metric heat map for E12 IgG binding to yeast displayed PTx S1-220 variants. Deep sequencing and entropy analysis of the variants selected from the IgG binding to a PTx S1-220 mutagenesis library implicated candidate residues (circled sections) predicted to be involved in binding. ***B,*** Validation of candidate residues identified from deep sequencing with yeast displayed PTx S1-220 variants. Candidate residues on PTx S1 predicted to be involved in binding were altered to the amino acid indicated. Binding of E12 IgG was assessed to the variant yeast displayed PTx S1-220 variants by ELISA. Data are represented as normalized binding relative to wild type (A450, variant/A450, wild-type), at an antibody concentration of 2 μg/ml, so that a lower value indicates reduced binding. Data shown are from an ELISA experiment with duplicate samples, and error bars represent the standard deviations of the mean.

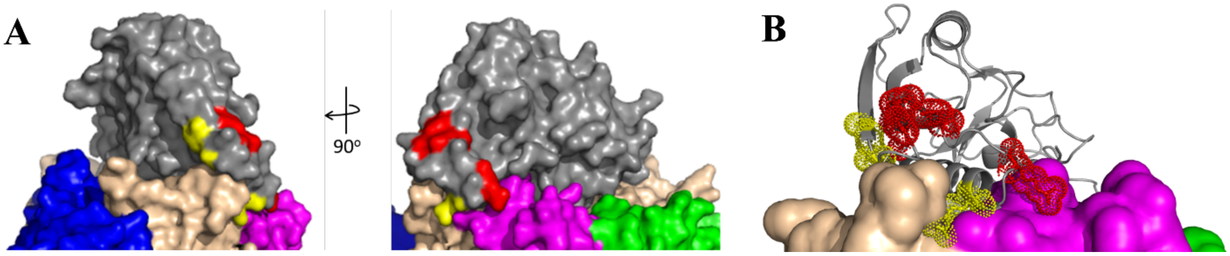

Figure S6. Comparison of A8 and hu1B7 epitopes on PTx-S1. ***A,*** Comparison of experimentally determined conformational epitopes. 90° Rotated views of the PTx S1 crystal structure (PDB 1PRT) with conserved residues indicated for A8 (yellow) and hu1B7 (red). ***B,*** Solvent accessible area around the key residues involved in A8 (yellow) and hu1B7 (red) binding to PTx S1 are represented by dots. The R76 residue involved in A8 binding appears less accessible between the S4 (tint) and S5 (magenta) subunits.

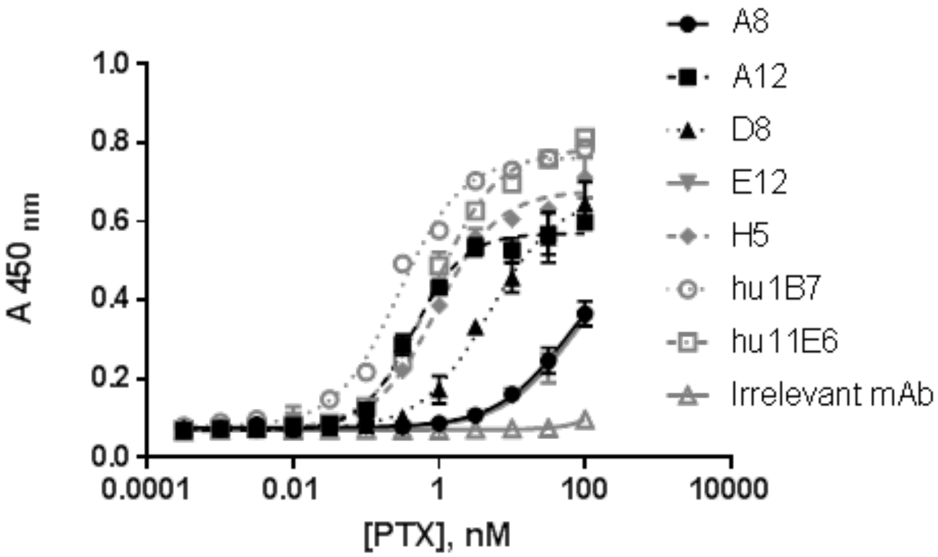

Figure S7. Antibodies bind receptor-bound PTx. The model receptor, fetuin, was coated onto ELISA wells at 10 μg/mL. After blocking, serially diluted PTx was added and allowed to equilibrate and then 1 μg/mL of the indicated antibody was added. Bound antibody was detected using secondary goat-anti-human HRP antibody. Error bars indicated are the standard error of the mean for duplicate antibody samples.

Table S1. Representative Luminex data: screening antibodies for PTx specificity.

**Average MFI**					
Sample #	Sample ID	PT	PT-A	PT-B	Fc
1	E12	138	874	15	10058
2	A8	81	584	15	7644
3	H5	1694	15	693	9465
4	D8	2868	12	98	6322
	Background	8	13	15	233

**Fold over background**					
Sample #	Sample ID	PT	PT-A	PT-B	Fc
1	E12	17	70	1	43
2	A8	10	47	1	33
3	H5	212	1	47	41
4	D8	359	1	7	27

Microspheres coupled with the PTx holotoxin, A-subunit or B-oligomer were placed in wells of 96 well microplates and incubated with supernatants derived from transfected HEK293 cells for 30 minutes. After washing, the microplates were then incubated with a PE conjugated goat anti-Fc antibody for another 30 minutes. After incubation, the microplates were washed, resuspended in assay buffer and read in a Luminex FlexMap 3D. Reactivities were scored based on values of fold over background. Samples with high fold over background reactivities to PTx or subunits are indicated as shaded boxes.

Table S2. Yeast display sorting statistics for A8 and E12 IgG binding from PTx S1-220 library.

	Tile Length (AA)	Sort Labeling Conditions (pM)	Events Collected for Binding Population	Percent Sorted (Display)	Percent Sorted (Binding)
PTxS1-A8 Tile 1	72	64	400,000	63.02%	7.56%
PTxS1-A8 Tile 2	74	64	400,000	66.89%	7.27%
PTxS1-A8 Tile 3	73	64	400,000	63.84%	7.63%
PTxS1-E12 Tile 1	72	140.5	400,000	57.58%	6.63%
PTxS1-E12 Tile 2	74	140.5	400,000	62.27%	7.57%
PTxS1-E12 Tile 3	73	140.5	400,000	63.11%	7.27%

The different tiles contain the PTxS1-220 libraries with mutations concentrated to the indicated regions. Tile1 comprises of mutations in positions 1-72, Tile 2 contains mutations in positions 73-147, and tile 3 has mutations in codons 148-220.
